# Perceptions of childhood asthma and its control among Malays in Malaysia: a qualitative study

**DOI:** 10.1038/s41533-020-0185-z

**Published:** 2020-06-08

**Authors:** Nursyuhada Sukri, Siti Nurkamilla Ramdzan, Su May Liew, Hani Salim, Ee Ming Khoo

**Affiliations:** 1 Department of Primary Care Medicine, Faculty of Medicine, University of Malaya, Kuala Lumpur, Malaysia; 2 Department of Family Medicine, Faculty of Medicine and Health Sciences, University Putra Malaysia, Selangor, Malaysia

**Keywords:** Paediatric research, Paediatric research

## Abstract

Children with poor asthma control have poor health outcomes. In Malaysia, the Malays have the highest asthma prevalence and poorest control compared to other ethnicities. We aimed to explore Malay children with asthma and their parents’ perceptions on asthma and its control. We conducted focus group discussions (FGD) using a semi-structured interview guide. Interviews were audio-recorded, transcribed verbatim and analysed thematically. Sixteen children and parents (*N* = 32) participated. The perception of asthma was based on personal experience, cultural and religious beliefs, and there was mismatch between children and parents. Parents perceived mild symptoms as normal, some had poor practices, raising safety concerns as children were dependent on them for self-management. Conflicting religious opinions on inhaler use during Ramadhan caused confusion in practice. Parents perceived a lack of system support towards asthma care and asthma affected quality of life. Urgent intervention is needed to address misconceptions to improve asthma care in children.

## Introduction

Asthma is a chronic disease that affects millions of children worldwide^1^. It was ranked among the top ten causes of disability-adjusted life years in children aged 5−14 years^2^. Children with poor asthma control have been shown to have poor health outcomes, impaired quality of life, and even death^2,3^.

Malaysia is a multiracial country with three major ethnic groups, Malays (54%), Chinese (25%) and Indians (8%)^4^. All Malays are Muslim by law while Chinese and Indians profess different religions including Hinduism, Buddhism, Taoism, Sikhism, and Christianity^5^. The prevalence of children with asthma below 18 years old is estimated at 7.1%^6^. It is highest in Malays (8.1%), followed by Indians (7.4%) and Chinese (4.3%). Asthma control was also found to be poorest in Malays (35.7%), followed by Indians (33.5%) and Chinese (10.6%)^7,8^.

Sociocultural factors have been reported to influence the understanding of asthma and self-management. Some perceive asthma as hereditary and others perceive it as contagious^9,10^. Mild asthma is often perceived as normal, despite symptoms indicating poor control^11^. Some parents and physicians tend to underestimate the severity of children’s asthma and overestimate their asthma control^12^. Asthma self-management is influenced by one’s perception of asthma^11^ which may vary between ethnic groups due to diverse culture^13,14^. For instance, during the fasting period, Muslims would adjust their medication to enable them to fast as some Muslims are taught the use of an inhaler invalidates the fast^15^.

Parents play an important role in asthma management in the early years of childhood before children can manage their asthma independently^16^. However, some parents have misconceptions regarding asthma and its medication such as fear of steroid side effects and dependence, and avoidance of physical activity to improve asthma control^12^, which could in turn influence children’s perception of asthma^17^.

In Malaysia, the Malays make up the ethnic majority and Malay children have the highest prevalence of asthma and the poorest asthma control among the three major ethnic groups^6^. This finding was different from other countries such as the United Kingdom and the United States where asthma morbidities affect more people from ethnic minority groups^18,19^. Few studies examine the perception of children about asthma and its control, as previous literature is concerned about the perceptions of asthma from adults and caregivers, especially in Malaysia. We are still unsure of the factors contributing to poor asthma control among them. Thus, we aimed to explore the perceptions of Malay children with asthma and their parents about asthma and its control, to understand the reasons for poor asthma control.

## Results

### Demographic profile of participants

A total of 32 participants (16 Malay children with asthma and 16 parents) took part in 11 focus group discussions (FGD). Table 1 shows the participants’ socio-demographic profile. All parents interviewed were mothers.

**Table 1:** Socio-demographic profile of participants.

Demographic profile of the Malay children	*N* = 16
Age range (year)	10–12
Gender	
Female	8
Male	8
Level of asthma control	
Well-controlled	3
Partly controlled	9
Uncontrolled	4

Demographic profile of the parents (all Malay)	*N* = 16
Age range (year)	33−51
Gender	
Female	16
Male	0
Education background	
Primary	1
Secondary	9
Tertiary	6

Five themes emerged from the analysis: (1) Understanding of asthma, (2) Understanding of asthma control, (3) Children depend on parents for self-management practices, (4) Unsupportive healthcare and school systems and (5) Effects of asthma on children and parents.

### Understanding of asthma

Children’s and parents’ understanding of asthma, e.g., asthma symptoms, trigger factor and natural history, mainly came from their experiences of living with the disease itself or children’s personal experience and parents’ observations of the children’s illness. Parents’ understanding was also influenced by their own experience of having asthma and from others.

I have asthma right because I know it myself, I know how my child feels when he gets it, when the asthma is coming, we know how it feels…. (FGD4P, M15, Malay, 44 years old, mother of C14, C14 had partly controlled asthma)

Children viewed asthma as breathlessness, coughing, and/or wheezing, and used terms such as “semput” (short-winded) and “lelah” (breathlessness pertaining to asthma) to describe asthma symptoms and used it interchangeably. They also used terms such as “my breathing stuck” and “gasping for air” to describe breathlessness. Some children experienced severe asthma as near-death experience or life-threatening. Some believed asthma is an inherited disease. Table 2 illustrates asthma triggers perceived by children and their parents.

Sometimes I cough and I can’t even breathe, it gets stuck (breathing)…..then your nose is blocked, and then your mouth, it’s hard… (breathing) (FGD6C, C16, male, 11 years old, uncontrolled asthma)

When I had asthma (exacerbation) I feel like my life is taken away…dying, dying… (FGD6C, C4, male, 12 years old, well-controlled asthma)

**Table 2:** Asthma triggers perceived by children and parents.

	Parents’ perceptions	Children’s perceptions
Environmental	Dust, haze, pollen, pet, sand, crowded place, bird droppings	Dust, haze, fur, smoke
Weather	Weather too hot or too cold	Nil
Chemicals	Fabric of new clothes, colouring from medication and food	Food colouring
Food	Junk food, vinegar, tamarind, seafood, preservatives, “cold food”	Ice cream
Fruit	Papaya, orange, fermented fruit	Nil
Drink	Syrup drink, icy drink, carbonated drink, milo drink, ice cream, orange juice concentrate	Cold drinks
Emotion	Crying, excitement	Excitement, laughter
Physical activities	Running, exhaustion	Running while laughing, excessive exercise, running, star jump, walking up and down the staircase, playing soccer
Infection	Flu, fever, “infectious cough”, sinus, upper respiratory tract infection	Fever

However, parents viewed asthma as a test from God and accepted it as their responsibility to deal with it. They felt grateful that asthma was not something more challenging to handle. Even though parents believed asthma is an inherited disease, some parents also thought asthma would resolve as the children grow up.

It is another test from God for me… I accept that only one of my children has asthma, I am grateful. God didn’t give me a more difficult test… (FGD2P, M7, Malay, 47 years old, mother of C6, C6 had well-controlled asthma)

Hmm maybe because he’s still young now (the reason for uncontrolled asthma), I heard my friends say that when he is grown up, (asthma) it will be okay. (FGD2P, M7, Malay, 47 years old, mother of C6, C6 had well-controlled asthma)

### Understanding of asthma control

Children and parents benchmarked asthma control based on their previous experience of symptoms. They regarded asthma control as good when symptoms were less than previously, and some parents accepted mild asthma symptoms, e.g., daily cough and limitation to physical activity as normal for a child with asthma. Thus, they perceived their children’s asthma is only mild; hence, there was no need to follow up or use preventer medication.

My child’s one(asthma) is mild….when he coughs, I know he is going to get asthma. But it can disappear again… (FGD4P, M16, Malay, 40 years old, mother of C15, C15 had well-controlled asthma)

However, children and parents were mismatched in terms of understanding of asthma control. In this example, the child felt her asthma was uncontrolled, while the mother felt the control was better.

I think my asthma is not okay… I can’t sleep at night…. (FGD3C, C8, female, 12 years old, uncontrolled asthma)

Since she has grown older, I think her asthma has improved…. (FGD2P, M9, Malay, 46 years old, mother of C8, C8 had uncontrolled asthma)

Some children and parents believed sports would improve asthma control; however, a few parents still limited their children’s activities even though they thought their children’s asthma control was good.

Mom and dad won’t let me play sport because they are afraid that I will get an asthma attack. (FGD4C, M11, male, 11 years old, uncontrolled asthma)

He wanted to run… he wants to enter sports, but the problem is right he cannot, he needs to understand… (FGD3P, M12, Malay, 42 years old, mother of C11, C11 had uncontrolled asthma)

### Children depend on parents for self-management practices

Most children depended on their parents to self-manage asthma, which ranged from the recommended practice of using inhalers, to the use of various complementary alternative medicine (CAM) and home nebuliser before seeking hospital treatment. However, some parents’ self-management practices could be dangerous, potentially leading to delay in asthma treatment during emergencies. Table 3 illustrates the list of CAM used for asthma reported by children and parents.

I will turn him upside down like a baby. I will hold his feet, his father will hold him at the bottom, I will smack his back, ha (emphasize) let him breathe like usual first, if that still doesn’t work, if he is too pale, we will turn him to a standing position and smack his back… if that doesn’t work, I will send him to the hospital… (FGD1P, M1, Malay, 40 years old, mother of a child with partly controlled asthma)

**Table 3:** List of CAM used for asthma reported by children and parents.

	Examples
Supplements	Vitamins, fish oil extract, transfer factor, chlorella
Traditional or herbal medicine	Medicated ointment (camphor- or menthol-based), bird’s nest, Ayurvedic medication, sea cucumber
Homoeopathy or naturopathy	Honey, warm water, dates, stevia, black cumin seeds (*Nigella sativa*), pomegranate juice, garlic
Spiritual/religious healing	Healing rituals, meditation
Others	Turn body over, induce vomiting, air-ioniser

Besides that, parents feared the use of asthma medication for a long duration would cause dependency.

I do not use the aerochamber (inhalers).. because I am afraid, he would be addicted, whenever he gets it he will want it (inhalers) because when he goes to the hospital to get oxygen, there is no reduction…. (FGD1P, M1, Malay, 40 years old, children with partly controlled asthma)

Besides, we also found mismatched perceptions between children and parents in terms of self-management. Children felt they are dying during the exacerbation; however, parents still felt confident to manage their child’s asthma.

When I have asthma, I feel like dying (FGD6C, C16, male, 11 years old, uncontrolled asthma)

So far all praise to God, no matter how bad it was, we could manage… (FGD1P, M2, Malay, 51 years old, mother of C16, C16 had uncontrolled asthma)

For self-management during the fasting month, parents will readjust the timing for medication according to the fasting timeline so that the children were able to fast during the day.

During the fasting month I will use inhalers after breaking the fast at night and also during sahur(dawn)….. (FGD4C, C11, male, 11 years old, uncontrolled asthma)

The use of inhalers during fasting month also raised concerns among children and parents whether it would invalidate the fast because there were different views among the Islamic scholars regarding the use of inhalers during the fasting month.

I don’t use it….I can’t… because of fasting (as it will invalidate fasting)…. (FGD5C, C13, male, 10 years old, well-controlled asthma)

There are different opinions although it is allowed because it doesn’t go into the (food) passageway, so when fasting, he can continue fasting…and they’re still kids….. (FGD1P, M3, Malay, 42 years old, mother of C1, C1 had well-controlled asthma)

Hmm about the article, hmm I read two. One said it cancels your fast, and another article said it doesn’t cancel your fast because it (use of inhalers) is a necessity…. (FGD4P, M16, Malay, 40 years old, mother of C15, C15 had well-controlled asthma)

### Unsupportive healthcare and school systems

Healthcare system and schools were perceived as unsupportive of asthma care. Some parents were dissatisfied with the treatment provided and the lack of continuity of care from health services. Others were unhappy that the healthcare providers were not attentive, and there was distrust of healthcare provision. Parents also complained follow-up appointments were not given after emergency treatment. Some children were bored by long waiting time at clinics during the follow-up and feared hospital admission.

Yes they don’t pay attention, that is correct…. when our child gets asthma he does not check, does not want to check his chest, breathing….nothing….. I don’t like these people, I don’t like them at all, I hate them, I hate the service that I get from them… (FGD1P, M1, Malay, 40 years old, mother of children with partly controlled asthma)

Children and parents also perceived a lack of help with asthma management at school. Children needed to self-manage asthma at school, and the teacher will only call parents to pick them at school when children started to feel uncomfortable due to asthma symptoms or when they have attacks. Children were also left alone at sickbay while waiting for the parent to fetch them.

… My teacher told me to use it (inhaler) outside the class to avoid disturbance…then after done taking the puff to get back into class and rest…. (FGD6C, M16, male, 11 years old, uncontrolled asthma)

### Effects of asthma on children and parents

Children and parents felt asthma had limited their daily life, including travelling on holiday and praying at a mosque. Some children felt asthma was a stigma and would not disclose asthma to others. Often, parents had to take leave from work to take care of their children when they had an exacerbation or were admitted to hospital. Some parents perceived asthma as costly if they sought treatment at private centres.

Hmm a little bit embarrassed, I got teased once…how did he say, hmm asthma boy… even if I bring my inhalers to school, I’ll do it secretly. (FGD6C, M16, male, 11 years old, uncontrolled asthma)

It is difficult to find a hotel without carpets…..there are many things that I need to avoid when on holiday…another thing is that if we go to the mosque, the carpet of the mosque.… (FGD2P, M7, Malay, 47 years old, mother of C6, C6 had well-controlled asthma)

Several parents were stressed and concerned about the effect of asthma on children’s future, such as staying in a boarding school.

Hmm for that I did feel very depressed because other people’s children are normal… they go to school like normal, play around… ha (emphasize) my daughter does not… (FGD1P, M5, Malay, 42 years old, mother of C2, C2 had well-controlled asthma)

## Discussion

Our findings highlight the importance for healthcare providers to address perceptions of asthma and its control not only from the parents’ point of view, but also the children’s, as they are the one who experienced the disease. Religious beliefs influenced the understanding of asthma and its management, especially on the use of inhalers during fasting month which was a concern among Malay children and parents. The mismatch of perceptions of asthma between children and parents could affect asthma outcome, e.g. poor asthma control, increased hospitalisation and quality of life. Children and parents were found to have poor understanding and misconception of asthma control, e.g. understanding normality in children and mismatch of perceptions of asthma control. Therefore, when parents had a mismatched and poor understanding of asthma control and the child is reliant on the parents, danger may arise because it may delay parents from seeking medical help. Negative experiences with the healthcare provider and school could also be a barrier for asthma care in children. The majority of the parents interviewed were female. This is the norm in Malay families where mothers mostly did the caregiving of children and fathers are usually the breadwinner for the family^20^.

Religious and cultural beliefs influence parents’ understanding of asthma. Some parents view asthma as a test from God as they viewed health as the greatest blessing that God had given to humankind and would accept such illnesses like asthma with patience, prayers, and meditation^21^. Malay parents were more ready to accept their children’s condition and even felt grateful about this. Some perceived that their children are not normal due to asthma, and a similar finding was found in other studies as well^21–24^. In non-Muslim countries, some qualitative studies showed that the mother would be in denial when their child was diagnosed with asthma and perceived asthma as a contagious disease^23,24^. Therefore, healthcare provider should be aware and addressed parents’ understanding of asthma taking into account their beliefs and culture during consultation.

Children were not expected to fast due to their young age until they reached puberty when fasting becomes an obligation. Even though most of them mentioned their asthma control had not been affected, readjusting medication time without discussion with a healthcare provider could affect asthma control and worsen the symptoms^25^. There was also confusion among parents and children regarding the use of inhalers during fasting months as guidelines on the use of the inhalers vary in different Muslim countries^15,26^. Some permitted use of inhalers while others regard it as breaking the fast during the fasting month. In non-Muslim countries, such as in the United Kingdom, children were advised to break the fast if asthma worsens^27^. Fasting is not specific to Islam, but this was not an issue in other religions as the use of medication during fasting month was not considered breaking the fast^28^. This confusion needs to be addressed, or it could contribute to non-adherence to asthma medication during the fasting month. A study showed that only 13% of adult asthma patients used medication as usual during the fasting month, while the remaining altered the timing of inhaler use^29^. The Islamic scholars and Ministry of Health could convene a consensus among them and issue a statement or guide to inhaler use during fasting month so that consistent message is delivered to Muslim patients with asthma.

We found there was a mismatch between children’s and parents’ perceptions of asthma. Even though both children and parents understood asthma from their experiences, children were the ones who experienced the symptoms. Children understood asthma mainly based on their experience of symptoms while the parents only observed the children and accepted it as a test from God. Rather than just observing the symptoms, parents should talk to the children about their asthma to help understand their condition better^30^. This discrepancy showed communication gap between children and parents, which could lead to poor asthma management in children^31^.

A mismatch was also found in the understanding of asthma control between children and parents where children felt their asthma control was uncontrolled while parents thought the child’s asthma was improving as the child grew up. This mismatch in perceptions was quite worrisome as it might influence parents in managing their child’s asthma^32^. Children and parents had a poor understanding of asthma control as they perceived mild symptoms as normal. Parents would restrict their children from physical activities to avoid getting asthma symptoms. These views were contrary to the aim of achieving good asthma control which is that children with asthma could have a normal life^33^. Reducing physical activity in children to prevent asthma symptoms could lead to detrimental effects such as the increased risk of obesity which may, in turn, worsen their asthma^34^. Other studies among different ethnic groups also found participants’ perception of asthma and its control were strongly influenced by their understanding of the disease^35^. Children’s perception of asthma control was influenced by the parents’ perceptions. Therefore, it is essential to educate children, and correct misconceptions before they grow into adulthood^36^ or the children will continue having misunderstanding about asthma and its control.

We found that poor perceptions and mismatch of asthma control led to poor self-management practices among parents. Some parents used dangerous methods for self-management, such as turning the child upside down during an asthma attack. This is worrisome as parents might not recognise asthma severity nor understand the danger of such action. This is similar to other studies^13,37^ whereby parents tried various ways to improve children’s asthma control, such as the use of CAM and the home nebuliser. Parents feared the use of preventer medication for asthma would cause side effects and dependency, a common concern in Asian and western countries^38–40^. However, the use of CAMs without proper monitoring and over-reliance on the use of nebuliser at home could potentially lead to delay in seeking treatment, causing morbidity and even mortality^41^. Our finding also showed that children felt they are dying during the exacerbation while parents felt they still can self-manage, which could bring more harm to the children. Therefore, the parent should be made aware of their limitations in self-management and seek medical attention appropriately^42^. Healthcare providers should be made aware of dangerous self-management practices which may be influenced by cultural and religious beliefs^43^. Parents and children also need to be educated on asthma and evidence-based management taking into account the cultural influence and myths^13^ as parents were the primary caregivers for the children until they reached the age of majority, which is 18 years old in Malaysia^44^ as children were reliant on parents’ self-management practices.

Children and parents viewed health services and schools as unhelpful towards asthma care. This was similar to findings from another study and appeared to be a barrier in achieving good asthma control in children^45^. A previous study found the negative experience with healthcare provider influenced health-seeking behaviour^46^. Children should be reviewed for asthma even if the asthma is mild, as it could recur in adulthood, and childhood asthma is a risk factor for later developing pulmonary disease^47^. The National Asthma and Education Program suggested that parents should inform the teacher about their children’s written asthma action plan to guide the teacher during emergencies rather than waiting for parents to fetch their children at school without taking immediate action that could save a life. Therefore, a future study could look into intervention at school to improve asthma knowledge and awareness to improve asthma outcome. For instance, a culturally tailored school-based intervention has been shown to reduce hospitalisation, reduce emergency department visits and improvement in days of restricted activity^48^.

This is one of the very few qualitative studies done among children with asthma. This study explored the views and practices of children with asthma and their parents including cultural aspects in Malay children who had the worst prevalence and asthma control among all ethnic groups in Malaysia. These findings would provide information for a culturally tailored intervention to improve asthma control, taking into account individual and family’s understanding of the disease and its control, the system barriers, culture and religious impact. This study was limited by a lack of recruitment of fathers and children younger than 10 years who refused to be interviewed.

In conclusion, the perception of Malay children on asthma and its control was influenced by parents, who were in turn were influenced by cultural and religious factors. There was a mismatch between children’s and parents’ perception, which could contribute to poor asthma care. Thus, parents and children must be educated on asthma, and its control as children depend on their parents for self-management. A culturally tailored intervention for children with asthma and their parents is needed to improve asthma care.

## Methods

### Identifying and recruiting children with asthma

We used a qualitative approach and recruited children from two suburban primary schools in Port Dickson district, Negeri Sembilan, Malaysia, between March and June 2016. A screening questionnaire was distributed to all pupils in each school for parents to complete to identify children with parents-reported physician-diagnosed asthma. We subsequently purposively sampled these Malay children of different ages (7–12 years). Informed consent was obtained from parents and assent from children to participate prior to the conduct of FGD. Separate FGDs were conducted for children and parents to facilitate group dynamics and interaction between the participants as well as to capture norms that persist in the community^49^.

### Regulatory approval

Ethics approval and permission to conduct this study were obtained from University Malaya Medical Centre Ethics Committee (No. 20155-1366), the Ministry of Education Malaysia, the Department of Education, Negeri Sembilan and the two schools involved.

### Data collection

We conducted six FGDs among children (up to four participants in each group, duration ranged between 20 and 30 min) and five FGDs among parents (up to five participants in each group, duration ranged between 20 and 60 min). An additional interview was conducted for two children, one from a previous FGD participant to clarify certain issues discussed and another child who was not able to attend a previously arranged FGD as he was unwell. After reading through the transcript, two parents were re-interviewed to clarify further their views on perceptions of asthma and its control. Socio-demographic data were collected using a self-administered questionnaire from children and parents. Asthma control was defined as Well, Partly and Uncontrolled asthma using the 2016 Global Initiative for Asthma Guideline (GINA)^50^. This is a four-item questionnaire assessing daytime and nocturnal symptoms, use of relievers, limitations of activities for the past 4 weeks. Participants’ asthma control was assessed prior to the interview.

A semi-structured topic guide was used to facilitate the FGDs. It was constructed based on the biopsychosocial model^51^ and literature review to facilitate FGD. The biopsychosocial model systematically considers biological, psychological, and social factors and their complex interactions in understanding health, illness, and healthcare delivery. It offers a holistic picture of the factors that influence the health outcomes of children with asthma and integrate social, psychological, and biological factors on relations between parents and children with asthma^52^. The topic guide explored the understanding of parents and children towards asthma and its control, and their practices and experiences in managing asthma. After the initial focus group, a minor change was made to the topic guide. We added a question on participants’ views on school teacher’s involvement in asthma care at school. (Topic guide, supplementary references).

All FGDs were conducted in the Malay language as per participants’ preference. We used pictures of asthma medication such as inhalers, spacers and nebuliser to facilitate recognition of medication and devices. The FGDs were conducted either at schools or a health centre and were scheduled after school hours to minimise disruption to school activities. All interviews were audio-recorded, and a note-taker was present in each FGD to assist with note-taking and documenting non-verbal expressions. The FGDs were conducted until data saturation was reached when no new themes emerged.

### Data analysis

Data collection and analysis were conducted concurrently in keeping with the iterative nature of qualitative research. All FGDs were transcribed verbatim and checked for accuracy. All researchers were fluent in Malay (the national language), and the transcripts were analysed in Malay to avoid loss of semantics. For the purpose of reporting, the transcripts were translated to English by experienced translators who were bilingual, and the translated transcripts were checked for accuracy by S.N.R and H.S. Three researchers (N.S., S.N.R. and H.S.) independently coded the first transcript before discussion ensued. Any discrepancies were discussed, and consensus reached to formulate the coding framework. N.S. read all transcripts repeatedly to familiarise herself with the data and coded all transcripts. Any emerging codes were discussed with the team and added to the coding framework. We used Nvivo software version 10 to facilitate analysis. A thematic approach was used to analyse the data^53^. The themes that emerged were discussed and finalised with all research team members (N.S., S.N.R., E.M.K., S.M.L).

### Reflexibility and interpretation

N.S. is a master’s student in health research. She does not have asthma, and none of her family members is known to have asthma. Her understanding of asthma was based on her reading, discussion with others, and personal assumptions drawn from her existing knowledge on asthma. All other research team members are primary care physicians. Results were discussed iteratively within the team for a balanced interpretation.

### Reporting summary

Further information on research design is available in the Nature Research Reporting Summary linked to this article.

## Supplementary information

Supplementary Information

Reporting Summary

## Data Availability

The data that support the findings of this study are available from the corresponding author upon reasonable request.

## Code availability

The codes from the findings of this study are available from the corresponding author upon reasonable request.
