# Supplementary material for: Perceptions of childhood asthma and its control among Malays in Malaysia: a qualitative study
Source: NPJ Prim Care Respir Med. 2020 Jun 8;30:26. doi: 10.1038/s41533-020-0185-z (PMC7280185; doi:10.1038/s41533-020-0185-z)
Supplement: Supplementary file 1 — Supplementary Information [file 41533_2020_185_MOESM1_ESM.pdf]

Childhood Asthma
Participant Interview Topic Guide (children)

Preamble:

- **Ice-breaking and explain that there is no right or wrong answer**
- **Explain need to get consent for the interview and audio-recording. Obtain consents.**
- **Explain that the participant does not have to answer if he or she does not wish to do so**

Question	Prompts
**Asthma experience**	
What do you know about asthma?	Experience? Reading? Told by someone?
Tell us about your asthma? How is it now?	What do you feel? Cough, nightime cough, difficulty breathing?
Does your asthma disturb you?	Activities affected e.g school? swimming, PJK, sleeping in fan?air conditioned room? Early morning or night bath? Play in the rain?
How do you know if it’s good or bad asthma?	Able to play? Easy breathing? No cough?
How does having asthma makes you feel?	Friends isolation/bully- because of illness, using inhalers.
**Asthma self-management**	
What do you do when you have your asthma?	Inhaler? Tell parents? Go to hospital/clinic? Asthma action plan?
Who take care of your asthma at home? School? Experience asthma at school?	You? Your parents? Maid? What do you/they do?
Do you take anything else other than these for your asthma? (showing inhalers and syrup)	Other medication from doctors?
**Asthma treatment**	
Do you take medicine? Tell us about your medicine.	Pills? MDI? Spacer? Aerochamber? Have you seen ‘these’ (peak flow meter) before? How do you use it? When do you use your medication? Do you need to take your medicine everyday? Which medication is better? Reliever ? controller? How would you know when do you need the pump?
How do you feel about taking medicines?	Dislike? Troublesome? Ashamed?
How often do you see the doctor for asthma?	Follow up? Emergency (hospital)? Stay in hospital ? When? Why?
When you go to clinic what was usually done?	Explanation? Medication?
What do you think about the clinic visits for asthma (clinic, doctor & medications)?	Difficult access? Language barrier? Waiting time? Unfriendly staff?

How do you feel when you come to clinic?	Unpleasant? Okay?
**Health belief about asthma**<br><br>Why do you think you have asthma?<br><br>What other things you do/eat for your asthma?<br><br>Do you have rules about asthma?	 <br>Inherited? Infection?<br><br>What are they? Home nebulizer? How does it help? Homeopathy? Which method is better for you? Why?<br><br>Food, drinks or activities? Any other rules? Who makes the rules? (rules at school and home)
**Source of information**<br><br>Where and from whom did you get information about asthma?<br><br>Anything else that you want to share about your asthma?	 <br>Clinic? Family? Friends? Internet? How good is the information?

THANK YOU

Childhood Asthma
Participant Interview Topic Guide (parents)

Preamble:

- **Ice-breaking and explain that there is no right or wrong answer**
- **Explain need to get consent for the interview and audio-recording. Obtain consents.**
- **Explain that the participant does not have to answer if he or she does not wish to do so**

Question	Prompts
**Asthma experience**	
What do you know about asthma?	Experience? Reading? Told by someone?
Tell us about your child's asthma? How is it now?	Cough, nightime cough, difficulty breathing?
Does your child's asthma disturb he/her? How?<br>Does your child asthma disturbs you? How?	Activities affected e.g school? swimming, PJK, sleeping in fan? air conditioned room? Early morning or night bath? Play in the rain?
How do you know if your child's asthma is good or bad?	Able to play? Easy breathing? No cough?
How does having asthma makes your child feel?	Friends isolation/bully- because of illness, using inhalers.
How does your child's asthma makes you feel?	Anxious? Overprotective?
**Asthma self-management**	
What do you do when your child has asthma?	Inhaler? Go to hospital/clinic? Asthma action plan?
Who take care of your child's asthma at home? School? What do you think on teacher involvement for asthma care?	You? Your child? Maid? What do you/they do?
Does your child take anything else other than these for his/her asthma? (showing inhalers and syrup)	Other medication from doctors?
**Asthma treatment**	
Does your child take medicine for asthma?<br>Tell us about your child's medicine.	Pills? MDI? Spacer? Aerochamber? Have you seen 'these' (peak flow meter) before? How do you use it? When do you use your medication? Do you need to take your medicine everyday? Which medication is better? Reliever ? controller?<br>How would you know when do your child needs the pump?
How do you feel about your child taking medicines?	Dislike? Troublesome? Ashamed?
How often do your child see the doctor for asthma?	Follow up? Emergency (hospital)? Stay in hospital ? When? Why?
When your child goes to clinic what was usually done?	Explanation? Medication?
What do you think about the clinic visits for asthma (clinic, doctor &	Difficult access? Language barrier? Waiting

medications)?<br><br>How do you feel when you accompany your child to clinic?	time? Unfriendly staff?<br><br>Unpleasant? Okay?
**Health belief about asthma**<br><br>Why do you think your child has asthma?<br><br>What other things you do/eat for your child's asthma?<br><br><br><br>Do you have rules about asthma?	 <br>Inherited? Infection? Environment?<br><br>What are they? Home nebulizer? How does it help? Homeopathy? Which method is better for you? Why?<br><br>Food, drinks or activities? Any other rules? Who makes the rules? (rules at school and home)
**Source of information**<br><br>Where and from whom did you get information about asthma?<br><br><br>Anything else that you want to share about your child's asthma?	 <br>Clinic? Family? Friends? Internet? How good is the information?
